# Stress and affective disorders in COVID-19 pandemic: On-line survey of russian respondents in different cities of residence

**DOI:** 10.1192/j.eurpsy.2021.785

**Published:** 2021-08-13

**Authors:** E. Kasyanov, G. Rukavishnikov, M. Sorokin, O. Makarevich, N. Neznanov, N. Lutova, G. Mazo

**Affiliations:** 1 Department Of Translational Psychiatry, Bekhterev National Medical Research Center for Psychiatry and Neurology, St. Petersburg, Russian Federation; 2 The Integrative Pharmaco-psychotherapy Of Mental Disorders, V.M.Bekhterev National medical research center for psychiatry and neurology, Saint-Petersburg, Russian Federation

**Keywords:** COVID-19, stress, affective disorders, pandemic

## Abstract

**Introduction:**

The psychological stress associated with the COVID-19 pandemic has a complex multifactorial nature.

**Objectives:**

The aim: to evaluate the level of stress during the COVID-19 pandemic in the Russian-speaking population with(-out) affective disorders in different cities of residence.

**Methods:**

The data obtained from an online survey of 4803 Russian-speaking respondents (age over 18) from March 30 to May 18, 2020. The survey included social, demographic and the history of affective disorders data. The anxiety distress level was evaluated with the Psychological Stress Measure (PSM-25).

**Results:**

Individuals from sub-cohort of Russian cities with populations less than one million had higher stress levels (M=135.39) compared to Moscow (M=129.47; p=0.003) or St.-Petersburg (M=126.63; p<0.001). However, stress scores in respondents with a history of affective disorders correspond to the average stress level according to PSM-25. Respondents without affective disorders from St.-Petersburg reported lower stress levels (M=92.88) than respondents from Moscow (M=100.47; p<0.001) and Russian cities with less than one million population (M=98.4; p<0.001). Average stress scores from St.-Petersburg and other Russian cities show a low level of stress on PSM-25, which indicates psychological adaptation. Stress scores from Moscow have borderline values between low and medium levels.

**Conclusions:**

Our study showed that the city of residence and affective disorders status significantly affect stress levels in Russian population. These factors could be further used in individual psychological support strategies.

